# The Synthesis and Functional Study of Multicolor Nitrogen-Doped Carbon Dots for Live Cell Nuclear Imaging

**DOI:** 10.3390/molecules25020306

**Published:** 2020-01-12

**Authors:** Yanan Zhang, Xingwei Zhang, Yanping Shi, Chao Sun, Nan Zhou, Haixia Wen

**Affiliations:** 1 Department of Physiology, Harbin Medical University, Harbin 150081, China; zhangyananzz678@163.com; 2 Department of Chemistry, Northeast Agricultural University, Harbin 150025, China; zhangxingwei123@aliyun.com (X.Z.); shiyanping160@aliyun.com (Y.S.); sscc961027@163.com (C.S.)

**Keywords:** nitrogen-doped carbon dots, multicolor illumination, multicolor cell nuclear imaging, mitosis

## Abstract

The nitrogen-doped carbon dots (N-CQDs) were synthesized by citric acid as a raw material and propylene diamine as a passivation agent. Structure, optical properties and biocompatibility of N-CQDs were analyzed. It was found that the N-CQDs possessed concentration-dependent, multicolor photoluminescence and low toxicity. As demonstrated in the imaging of bioluminescence, by adjusting the concentration of N-CQDs, the cell imaging effect can be adjusted. The internalized N-CQDs were concentrated in the nucleus. A novel tool for studying the nuclear changes during the cell cycle was developed.

## 1. Introduction

The nucleus is a vital component of cells [1], including a large majority genetic materials of cells and is involved in cell metabolism and heredity. The nucleus contains membraneless organelles, such as nucleoli and nuclear spots, which are highly organized and dynamic during the cell cycle [2]. As for the structure, how to form, why can it form and how to achieve biological function has become the focus of current research. Fluorescence imaging of the nucleus was the important tool to investigate these problems.

In the recent years, organic small molecule fluorescent substance related to nucleus imaging was widely investigated. However, as the development of study, numerous shortcomings were exposed, such as low selectivity, short excitation wavelength, low biocompatibility and so on [3]. Furthermore, DNA probe used in nucleus imaging usually has a high price, long synthesis time and low sensitivity [4]. These shortcomings restricted tremendously their further application in dynamic study of cellular physiological processes. In order to solve these problems, it is urgent to develop a novel probe.

In recent years, owing to the excellent biocompatibility, optical stability and easy surface modification, fluorescent carbon quantum dots have received intense attention in the biomedical field [5,6] and have more extensive application, especially in the biological imaging field [7,8,9,10]. Carbon quantum dots are usually smaller than 10 nm in size. Generally speaking, particles, with a size below 9 nm, can utilize the nuclear pore complexes enter into nucleus by free diffusion [11]. Interestingly, in numerous literatures, carbon quantum dots can only focus in the cytoplasm and are difficult to enter the nucleus. Therefore, only few research reports related to nucleus imaging.

At present, literature related to carbon quantum dots nucleus imaging can be divided into two categories. One is that employing biomass as raw materials to synthesize carbon quantum dots directly, and integrating into the cell nucleus and imaging, such as grape seeds, petals and bovine pancreatic ribonuclease [12,13,14]. This always happened by accidents, and still has no definite mechanism. The other is that by modifying actively the surface of carbon quantum dots, it can prompt it to integrate into the cell nucleus. This kind of literature can be divided into two strategies. One strategy is that a positively charged nitrogen atom is introduced onto the surface of the carbon quantum dots, such as quaternary ammonium [15,16]. The other is that constructing an amphoteric surface on the surface of carbon quantum dots, such as amino and carboxyl groups were introduced at the same time and control the proper surface charge by adjusting the ratio of them [17,18]. Other carbon quantum dots that can achieve nucleus imaging were also possessed such amphoteric surface [12,19,20]. This approach is relatively easy to achieve.

Taking account into the previous study, we prepared fluorescent carbon quantum dots by citric acid as a raw material and propanediamine as a passivation agent. It should be pointed out that a carboxyl and chain amino on the surface of the nitrogen-doped carbon dots (N-CQDs) constitutes an amphoteric surface. Analyzing the structure, photoluminescence and biocompatibility of the N-CQDs was successfully employed to the study of cell imaging.

## 2. Materials and Methods

### 2.1. Materials

Citric acid (CA), and propylenediamine (PDA) were bought from Aladdin Chemical Reagent Co. Ltd. (Shanghai, China). All the chemicals mentioned above are analytical grade and used directly. Ultrapure water was prepared from a Milli-Q water purification system (Millipore, Billerica, MA, USA) was used throughout the experiments. HeLa-229 and HCerEPic were obtained from the Cell Biology of Zhong Qiao Xin Zhou Cell Research and Tong Pai Cell Research (Shanghai, China). Fetal bovine serum (FBS) was bought from Shanghai Sango Biotechnology co., Ltd, CLARK, USA. Streptomycin and penicillin were bought from HyClone (Logan, USA).

### 2.2. Apparatus

The fluorescence measurements were obtained by a LS-55 fluorescence spectrophotometer (PerkinElmer, USA). The UV-vis spectra were recorded using a UV-2550 spectrophotometer (Shimadzu, Japan). The FT-IR spectra were obtained by a Magna-IR560 (Nicolet Co., Madison, WI, USA). The X-ray photoelectron spectroscopy (XPS) spectra were recorded using an AXIS Ultra DLD spectrometer (Kratos, Manchester, UK). Biological cells are imaged using an upright fluorescence microscope (Nikon Eclipse E800, Tokyo, Japan) and a laser confocal microscope (Carl zeiss LSM 800, Jena, Germany).

### 2.3. Synthesis of N-CQDs

For the preparation of N-CQDs, 3 g CA and 1.5 mL of PDA were dissolved in 28 mL ultrapure water to form a homogeneous solution. Then, the mixture was transferred to a poly(tetrafluoroethylene) (Teflon)-lined auto-clave (50 mL). Subsequently, the aqueous solution was heated at 180 °C for 5 h. After being cooled down to room temperature, the obtained black-brown dispersion was dialyzed against ultra-pure water through a dialysis membrane (300 Da) for 24 h to remove unreacted raw materials and low molecular weight by-products. Then, the water was removed by lyophilizing for 24 h in a vacuum freeze dryer. The powder was used for further characterization.

### 2.4. Cell Culture

Two cell lines, HeLa-229 and HCerEPic were obtained from the Cell Biology of Zhong Qiao Xin Zhou Cell Research (Shanghai, China) and Cell Biology of the America Sciencell (USA). The cells were cultured in 1640 supplemented with 15% fetal bovine serum (FBS) (Shanghai sango biotechnology co., Ltd, CLARK, USA). Of streptomycin 100 μg/mL and 100 units/mL of penicillin (HyClone, Logan, USA) was used. The cells were digested with 0.25% Trypsin-EDTA, 1000 RPM centrifugal 5 min, add 4 mL of medium in a humidified atmosphere with 5% CO_2_ at 37 °C. Fresh medium was replaced every 2–3 days when confluence reached approximately 80%–90%.

### 2.5. Cell Viability

Cell viability was measured using the Cell Counting Kit 8 (CCK8, Dalian Meilun Biotechnology Co., Ltd, China) according to the manufacturer’s instructions. In brief, cells were seeded in 96-well plates at a density of 5 × 10^3^ cells/well in 100 μL of medium. Then, the next day, HeLa and HCerEPic cells preloaded with plates were added with N-CQDs of different concentrations. On the third day, the culture medium containing 10% CCK-8 was directly prepared, which was added in the form of liquid exchange and incubated for 1 h in the incubator. The optical density (OD) was measured at 450 nm in a spectrophotometer.

### 2.6. Immunofluorescence

HeLa cells and HCerEPic were taken out of the culture box for pancreatic enzyme digestion, and then the pancreatic enzyme was absorbed. Appropriate medium was added to terminate the pancreatic enzyme digestion, and the cells were suspended by blowing and beating with a pipette. Centrifugation was done at 1000 rpm for 5 min. We poured out the clear liquid, by adding 13 mL of culture to beat the cell into a single suspension cells, with six orifices to join 2 mL per hole cell suspension culture cells, and then put in 37 °C and 5% CO_2_ cultivation in the box. After the cells were adhered to the wall, cells were cultured for 24 h and observed with fluorescence microscopy by adding N-CQDs of different concentrations. 

### 2.7. Statistical Analysis

The data were assessed using SPSS 18.0 software (IBM, SPSS, Chicago, IL, USA). All of the experiments were performed in triplicate, and Student’s *t*-test was used to analyze the significance of the different levels between two groups. Survival curves were estimated by the Kaplan–Meier method and compared using the log-rank test. The Cox proportional hazards model was used for univariate and multivariate analyses. The data are expressed as the mean ± standard deviation (SD) and differences of *p* < 0.05 were considered to be statistically significant.

## 3. Results and Discussion

### 3.1. Characteristics of N-CQDs

**Figure 1:**
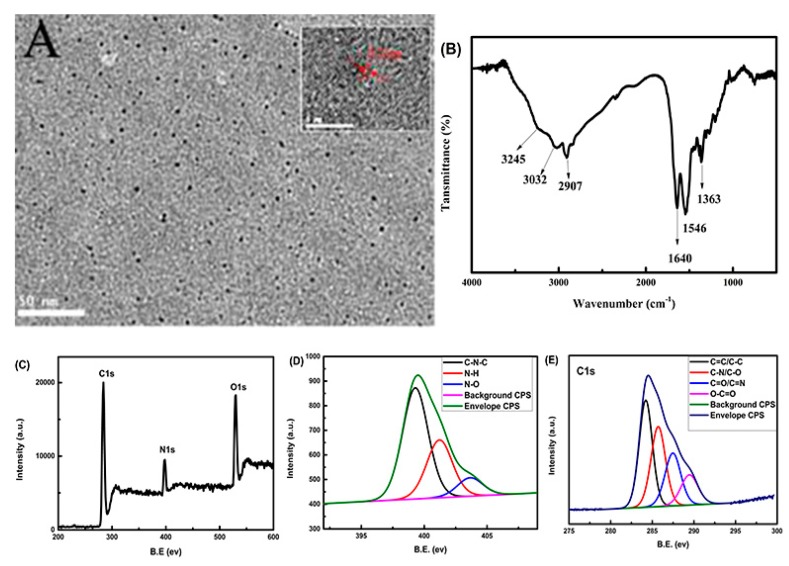
(**A**) TEM image of nitrogen-doped carbon dots (N-CQDs) (insert: local lattice amplification of single N-CQDs); (**B**) the FT-IR spectrum of N-CQDs; (**C**) the full-scale X-ray photoelectron spectroscopy (XPS) spectrum of N-CQDs; (**D**) N1s and (**E**) C1s spectra of N-CQDs.

As shown in Figure 1A, we found that the obtained products’ average diameter was about 5 nm. The HRTEM results revealed N-CQDs with a lattice spacing of 0.27 nm (the insert of Figure 1A).

As is shown in Figure 1B, the FT-IR spectrum showed a broad peak between 3020 and 3300 cm^−1^, which was attributed to N–H and O–H stretching vibrations. The intense peak at 1640 cm^−1^ could be assigned to C=O. The peak at 1363 cm^−1^ was due to the stretching vibration of C=N. The stretching and blending vibrations of –CH_2_^−^ were observed according to the distinctive peaks at 2907 and 2851 cm^−1^, separately. The XPS full scan spectrum (Figure 1C) showed three dominant peaks at 284.6, 399.5 and 531.45 eV, which was ascribed to C1s, N1s and O1s, respectively. The analysis of XPS showed that the CDs were mainly composed of 79.28% (C), 7.54% (N) and 13.19% (O). The fitting of the N1s spectrum (Figure 1D) showed three peaks at 399.3, 401.2 and 407.3 eV, which confirmed the existence of C–N–C, N–H and N–O. The C1s spectrum (Figure 1E) showed four peaks at 284.2, 285.7, 287.5 and 289.5 eV, indicating the presence of four types of carbon bonds: C=C/C–C, C–N, C=O/C=N and O–C=O. The analysis of FT-IR and XPS indicated that the obtained N-CQDs’ surface had C=O, C=N and –NH_2_, which proved that N atoms were successfully doped into N-CQDs.

In the procedures, usually the carboxyl groups of citric acid firstly polymerized with amine or hydroxyl groups to form amide or ester polymers, then the polymers were carbonized further to produce N-CQDs. With carboxyl group on the surface of N-CQDs, the amino group at one end of diaminopropane can form the amide structure, and the chain amino structure was formed on the other end. The N-CQDs amphoteric surface formed by carboxyl and amino group, which is conductive to the entry of it into the cell [21].

### 3.2. Optical Properties of N-CQDs

**Figure 2:**
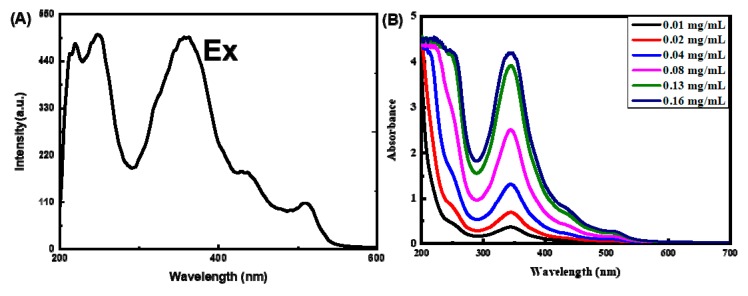
(**A**) The excitation spectrum of the N-CQDs and (**B**) ultraviolet spectrum of different concentrations of N-CQDs.

As shown in Figure 2A, the excitation spectrum revealed 4 excitation peaks at 248 nm, 357 nm, 430 nm and 510 nm, respectively. Showing the characteristics of multicolor luminescence. The four excitation peaks were independent, which was different from the multicolor fluorescence caused by the quantum size effect. The Figure 2B showed that the UV-vis spectra of N-CQDs with different concentration. The UV-vis spectra showed two absorption peaks at 250 nm and 344 nm in low concentration, consistent with the results obtained in excitation spectrum. The UV absorption 430 nm and 510 nm started to appear with increasing concentration and the intensity of two peaks were increased with increasing of concentration. The similar characteristics of absorption and fluorescence were obtained from citric acid as a raw material with a different method of preparation.

Most of the emission wavelength of N-CQDs displayed a marked excitation wavelength-independent character, the color of fluorescence emission was blue and green. The excitation wavelength at 200–250 nm might be due to the presence of surface Schiff base on the N-CQDs, while the excitation wavelength at 350–400 nm might be attributed to the surface amide groups on the N-CQDs, which is similar to the most of references [22].

**Figure 3:**
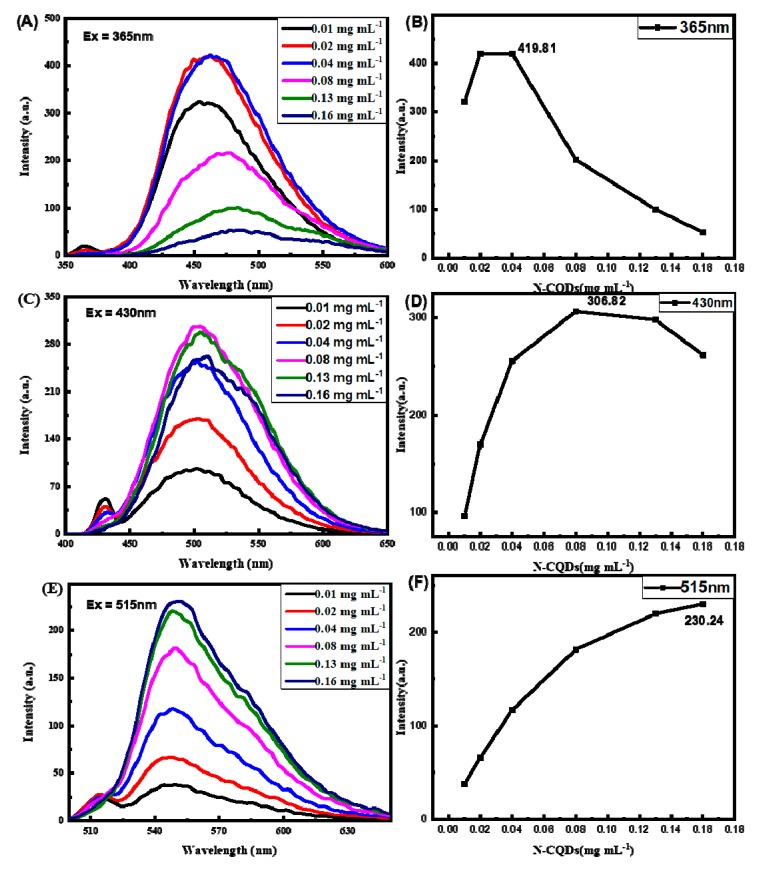
(**A**,**C**,**E**) Emission spectra excited with 365 nm, 430 nm and 515 nm light as a function of N-CQDs concentration, respectively and (**B**,**D**,**F**) fluorescence intensity of N-CQDs with different excitation wavelengths at 365 nm, 430 nm and 515 nm, respectively.

In order to identify the mechanism of the nature of two peaks, we studied the intensity of emission peaks at three excitation wavelengths with different concentration of N-CQDs. As shown in Figure 3A, at emission peaks of 470 nm with the excitation wavelength at 365 nm. Due to the inner filter effect the intensity of fluorescence enhanced rapidly to the maximum and then declined rapidly with the increasing of concentration (Figure 3B). At emission peaks of 500 nm and 550 nm with the excitation wavelength at 430 nm (Figure 3C) and 515 nm (Figure 3E), respectively, the intensity of fluorescence greatly enhanced with the increasing of concentration (Figure 3D,F). The intensity of emission was higher than excitation of 365 nm at high concentration. The changing of concentration could affect the interactions between C-dot nanoparticles on their fluorescence emission properties: wavelength and intensity modifications. It may be due to the surface being rich in –NH_2_ and –COOH on N-CQDs. Some of the N-CQDs were connected and formed the structure like amino acid inner salt when the concentration reached a level. The emission peaks red-shifted due to the extended conjugation in the C-dot@C-dot structure [23]. Therefore, the fluorescent color of N-CQDs could easily be adjusted by changing the concentration of N-CQDs.

### 3.3. Cytotoxicity

**Figure 4:**
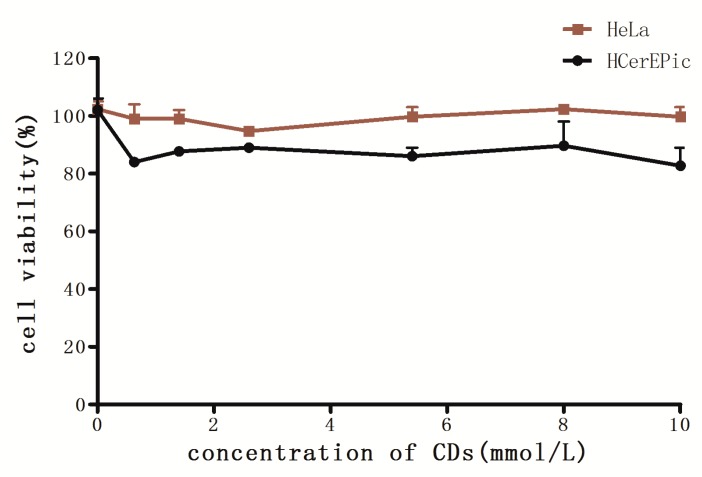
Cell Counting Kit 8 (CCK-8) assay was used to examine the effects of N-CQDs with different concentrations on the survival rate of two kinds of cells.

The labeling of cells is of great significance in the field of biomedical for understanding their division, migration and the formation process and function of organelles. Thus, there are high requirements for the biocompatibility of cell dyes. For this reason, we tested the toxicity of N-CQDs on cells using CCK-8 assay. As shown in Figure 4, the result showed that no matter for normal cells or cancer cells, N-CQDs always possessed low cytotoxicity and good biocompatibility. It is proved that the as-prepared N-CQDs could be used for the labeling of living cells and dynamic study of physiological processes.

### 3.4. Multicolor Cell Imaging

**Figure 5:**
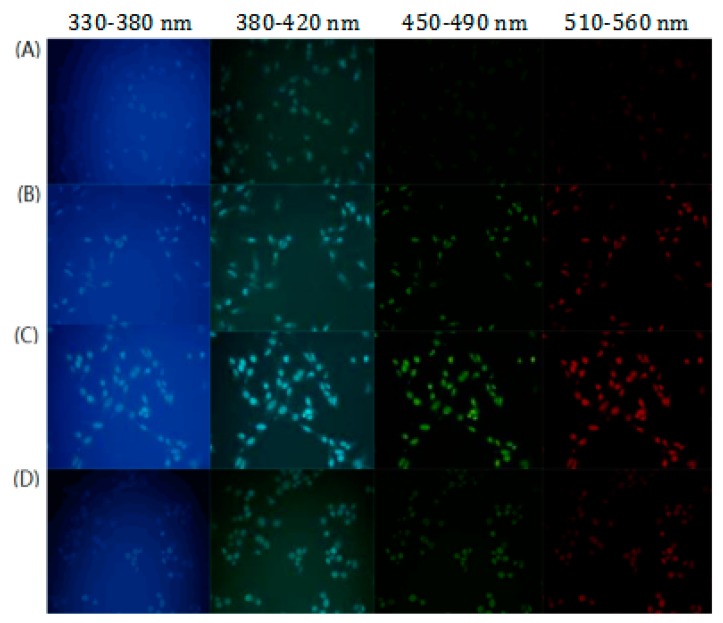
(**A**–**C**) Fluorescent images of living Hela cells with different concentration of N-CQDs (0.8 mM, 1.354 mM and 1.6 mM) at varied excitation wavelength. (**D**) Fluorescent images of living HCerEPic cells with N-CQDs (1.6 mM) at varied excitation wavelength.

The N-CQDs we synthesized possess concentration-dependent multicolor fluorescence emissions are the most prominent feature. This feature shows great potential in applications such as molecular imaging and in vivo molecular tracking. Firstly, we compared the imaging effects of N-CQDs at different concentrations towards Hela cells, and then the imaging effects of N-CQDs with same concentrations towards cancer cells and normal cells, the results shown in Figure 5, the results indicate that N-CQDs exhibit the characteristic of multicolor luminescence, it presents blue, cyan, green and red light respectively under varied excitation irradiation. However, green and red light could not present at a low concentration. With the increase of concentration, the intensity gradually increased from scratch, and showing an obvious concentration dependence. This characteristic is contributed to estimating the changes of concentrations of N-CQDs in cells and tracking the process of cellular proliferation.

Moreover, the comparison showed that the imaging effects of normal cells and cancer cells were significantly different under the same concentrations of N-CQDs. In particular, green and red light of normal cells was obviously inferior to cancer cells. The possible reason is that the permeability of cancer cells is usually higher than that of normal cells, and N-CQDs in cancer cells possess a higher concentration under the same conditions. This property could be potentially applied to distinguish various types of cells or flow cytometry.

### 3.5. Cell Imaging

**Figure 6:**
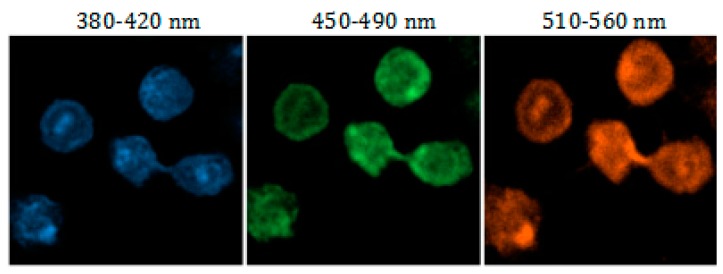
Laser confocal image of living Hela cells with multicolor cell imaging of interphase.

Cells staining with N-CQDs. Hela cells were observed by fluorescent microscope, it was found that N-CQDs were located in the nucleus and presented the effect of multicolor luminescence (Figure 6). Only weak blue and cyan light presented in the cytoplasm, and almost no fluorescence intensity of red and green light. The 1.6 mM CDs were added to the cells and incubated for 24 h. It is indicated that N-CQDs would be concentrated in the nucleus automatically after entering the cell membrane, and the concentration in the cytoplasm was very low.

Cells staining with N-CQDs. Hela cells were observed by fluorescent microscope, it was found that N-CQDs were located in the nucleus and presented the effect of multicolor luminescence (Figure 6). almost no fluorescence intensity of blue green and red light in the cytoplasm. The 1.6mM N-CQDs were added to the cells and incubated for 24 h. It is indicated that N-CQDs will be concentrated in the nucleus automatically after entering the cell membrane, and the concentration in the cytoplasm is very low.

**Figure 7:**
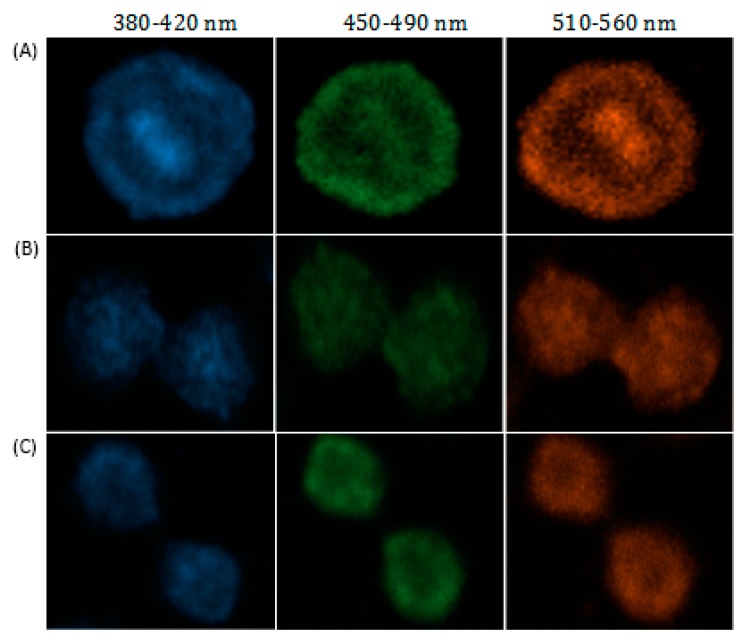
(**A**–**C**) Asynchronous cell imaging shows mitotic cells stained by N-CQDs visualizing chromosome aggregation through progression of mitosis’ prophase, metaphase and anaphase, respectively. The 1.6 mM N-CQDs were added to the cells and incubated for 24 h.

As shown in Figure 7, asynchronous cell imaging shows mitotic cells stained by DSF visualizing chromosome aggregation through progression of mitosis. Further, N-CQDs Laser confocal microscope imaging also visualizes characteristic structural changes in cells as cells progress through the cell cycle. As shown in the fluorescence picture (Figure 7A) of a single cell, when asynchronous cells are imaged, the majority of labeling cells are in interphase, whereas cells undergoing mitotic phases were also obviously observed. As shown in higher resolution micrographs (Figure 6) of a single cell, when asynchronous cells were imaged, the majority of labeled cells were in interphase but cells undergoing mitotic phases were also clearly observed. The internalized nitrogen-doped carbon dots were enriched in the nucleus and combined with chromatin, light intensity varied with the concentration of chromatin as well.

## 4. Conclusions

N-CQDs were synthesized by using citric acid as raw material and propanediamine as passivator. It appeared that carboxyl group and chain amino group were introduced on the surface of N-CQDs. The aqueous solution of nitrogen-doped carbon dots showed concentration-dependent multicolor photoluminescence. Furthermore, these nitrogen-doped carbon dots entered the cells and showed no remarkable cytotoxicity. Different imaging effects can be obtained in the process of cellular imaging by adjusting the concentrations of different quantum dots. The internalized nitrogen-doped carbon dots were enriched in the nucleus and combined with chromatin, which exhibited that light intensity varied with the concentration of chromatin. This could be a new way for tracking individual cells or visualizing processes such as mitosis based on nucleus labeling. New tools are provided for tracking chromatin phase changes during cell cycle changes.
